# Prognostic impact of immune-related adverse events on patients with and without cardiovascular disease: a retrospective review

**DOI:** 10.1186/s40959-021-00112-z

**Published:** 2021-07-06

**Authors:** Shingo Kazama, Ryota Morimoto, Yuki Kimura, Naoki Shibata, Reina Ozaki, Takashi Araki, Takashi Mizutani, Hideo Oishi, Yoshihito Arao, Tasuku Kuwayama, Hiroaki Hiraiwa, Toru Kondo, Kenji Furusawa, Tomoya Shimokata, Takahiro Okumura, Yasuko K. Bando, Yuichi Ando, Toyoaki Murohara

**Affiliations:** 1 Department of Cardiology, Nagoya University Graduate School of Medicine, 65 Tsurumai-cho, Showa-ku, Nagoya, Aichi 466-8550 Japan; 2 Department of Oncology and Chemotherapy, Nagoya University Hospital, Nagoya, Japan

**Keywords:** Immune checkpoint inhibitors, Immune-related adverse events, Cardiotoxicity, Prognosis, Cardiovascular history

## Abstract

**Background:**

The emergence of immune checkpoint inhibitors (ICIs) has brought about a paradigm shift in cancer treatment as the use of these drugs has become more frequent and for a longer duration. As a result of T-cell-mediated inflammation at the programmed cell death-1, programmed death-ligand-1, and cytotoxic T-lymphocyte antigen-4 pathways, immune-related adverse events (irAEs) occur in various organs and can cause a rare but potentially induced cardiotoxicity. Although irAEs are associated with the efficacy of ICI therapy and better prognosis, there is limited information about the correlation between irAEs and cardiotoxicity and whether the benefits of irAEs apply to patients with underlying cardiovascular disease. This study aimed to investigate the association of irAEs and treatment efficacy in patients undergoing ICI therapy with and without a cardiovascular history.

**Methods:**

We performed a retrospective review of the medical records of 409 consecutive patients who received ICI therapy from September 2014 to October 2019.

**Results:**

Median patient age was 69 years (29.6% were female). The median follow-up period was 278 days. In total, 69 (16.9%) patients had a history of any cardiovascular disease and 14 (3.4%) patients experienced cardiovascular irAEs after ICI administration. The rate of cardiovascular irAEs was higher in patients with prior non-cardiovascular irAEs than without. The prognosis of patients with irAEs ( +) was significantly better than that of the patients without irAEs (*P* < 0.001); additionally, this tendency did not depend on the presence or absence of a cardiovascular history. Furthermore, the Cox proportional hazards analysis revealed that irAEs were an independent predictor of mortality.

**Conclusions:**

Although cardiovascular irAEs may be related to prior non-cardiovascular irAEs under ICI therapy, the occurrence of irAEs had a better prognostic impact and this tendency was not affected by cardiovascular history.

**Supplementary Information:**

The online version contains supplementary material available at 10.1186/s40959-021-00112-z.

## Background

Emergence of immune checkpoint inhibitors (ICIs) has led to a paradigm shift in cancer treatment as their use continues to expand [1, 2]. ICI administration has substantially improved clinical outcomes across a range of cancer types, particularly in malignant melanoma, non-small cell lung cancer, and renal cancer. ICIs suppress immune reactions, but their suppressive mechanism can be decreased by administering inhibitory antibodies against these molecules [1]. This enhances the body’s natural immune response to cancer, allowing it to kill cancer cells. Conversely, activated T-cell responses may not be specific to cancer cells and might target normal tissue, leading to immune-related adverse events (irAEs) [3]. IrAEs have reportedly occurred in nearly all organs and are particularly common in non-cardiovascular organs such as the colon, lungs, endocrine glands, skin, and liver. Recently, several studies have reported that the development of irAEs was associated with clinical benefits for patients receiving ICI therapy [4–6]. Meanwhile, cancer and cardiovascular diseases often coexist because of shared risk factors such as hypertension, obesity, smoking, and diabetes [7], and multiple types of anti-cancer drugs and therapies can cause cardiovascular disorders. When accelerated T-cell-mediated inflammation in the cardiovascular system occurs during ICI therapy, cardiotoxicity associated with ICI treatment such as myocarditis, vasculitis, pericardial diseases, left ventricular systolic dysfunction, rhythm disorders, and acute coronary syndrome occurred infrequently [8–15]; moreover, myocarditis in particular can lead to fatal consequences [16]. Although the dense myocardial capillary network that interacts with immune cells and the myocardium is susceptible to immune reactions with the onset of non-cardiovascular irAEs [17], little is known about the relationship between non-cardiovascular irAEs and cardiotoxicity. Furthermore, it is unclear whether the occurrence of irAEs is a good prognostic factor even in the presence of cardiovascular disease in patients receiving ICI therapy. Therefore, we performed a detailed investigation of irAEs including the presence of cardiotoxicity under ICI therapy and the correlation between irAEs and prognosis with and without a history of cardiovascular disease.

## Methods

### Study design and population

We retrospectively reviewed the medical records of consecutive patients who received their first ICI administration from September 2014 to October 2019. All patients who received ICIs were enrolled. In principle, ICIs were used for cancers in which the primary lesion was unresectable and for recurrent or irreversible cancers. Patient characteristics before ICI administration and the subsequent prognosis or occurrence of adverse events were evaluated. We obtained prior approval that the study protocol conformed to the ethical guidelines of the 1975 Declaration of Helsinki from the Ethics Committee of Nagoya University Hospital (approval number 2019–0176).

### Type of ICI

The ICIs were chosen from the following types: programmed cell death-1 (PD-1) inhibitors (nivolumab or pembrolizumab), programmed death-ligand-1 (PD-L1) inhibitors (atezolizumab or durvalumab), and cytotoxic T-lymphocyte antigen-4 (CTLA-4) inhibitors (ipilimumab). The type and dose of ICI had been determined by the attending physician of each department, with some administered as part of a combination therapy.

### Definition of non-cardiovascular and cardiovascular irAEs

The irAEs were diagnosed according to the American Society of Clinical Oncology (ASCO) guidelines [18]. According to medical records, patients who had clearly been described as experiencing an irAE of grades 2–4, as defined in the ASCO guidelines, were considered to have developed irAEs. Cardiovascular irAEs included myocarditis, pericarditis, acute coronary syndrome, arrhythmia, heart failure, vasculitis, and venous thromboembolism with over mild-to-moderate symptoms post ICI therapy.

### Definition of cardiovascular history

Cardiovascular history was defined as the presence of a prior diagnosis of a cardiovascular disease before ICI administration, such as with coronary artery disease, heart failure, arrhythmia, venous thromboembolism, and pericardial disease.

### Outcome

The primary outcome was the occurrence of all-cause mortality within the follow-up period.

### Statistical analysis

Continuous data were presented as medians with interquartile ranges (IQRs) and were compared with the Mann–Whitney U test. Categorical variables were expressed as counts (percentages) and were compared with the Chi-square test or Fisher’s exact test. Kaplan–Meier curves and the log-rank test were used to quantify the relationship between the occurrence of irAEs and the survival rate. The Cox proportional hazards regression analysis was performed to identify the predictors of all-cause mortality. Additionally, a landmark analysis at 3 months after initiating ICI treatment was performed to adjust for the effects of early progression or death, in which patients who experienced events in the first 3 months were excluded. All statistical analyses were performed using R version 3.5.2 (R Foundation for Statistical Computing, Vienna, Austria), and statistical significance was accepted for *P*-values < 0.05.

## Results

### Patient characteristics at baseline

**Fig. 1:**
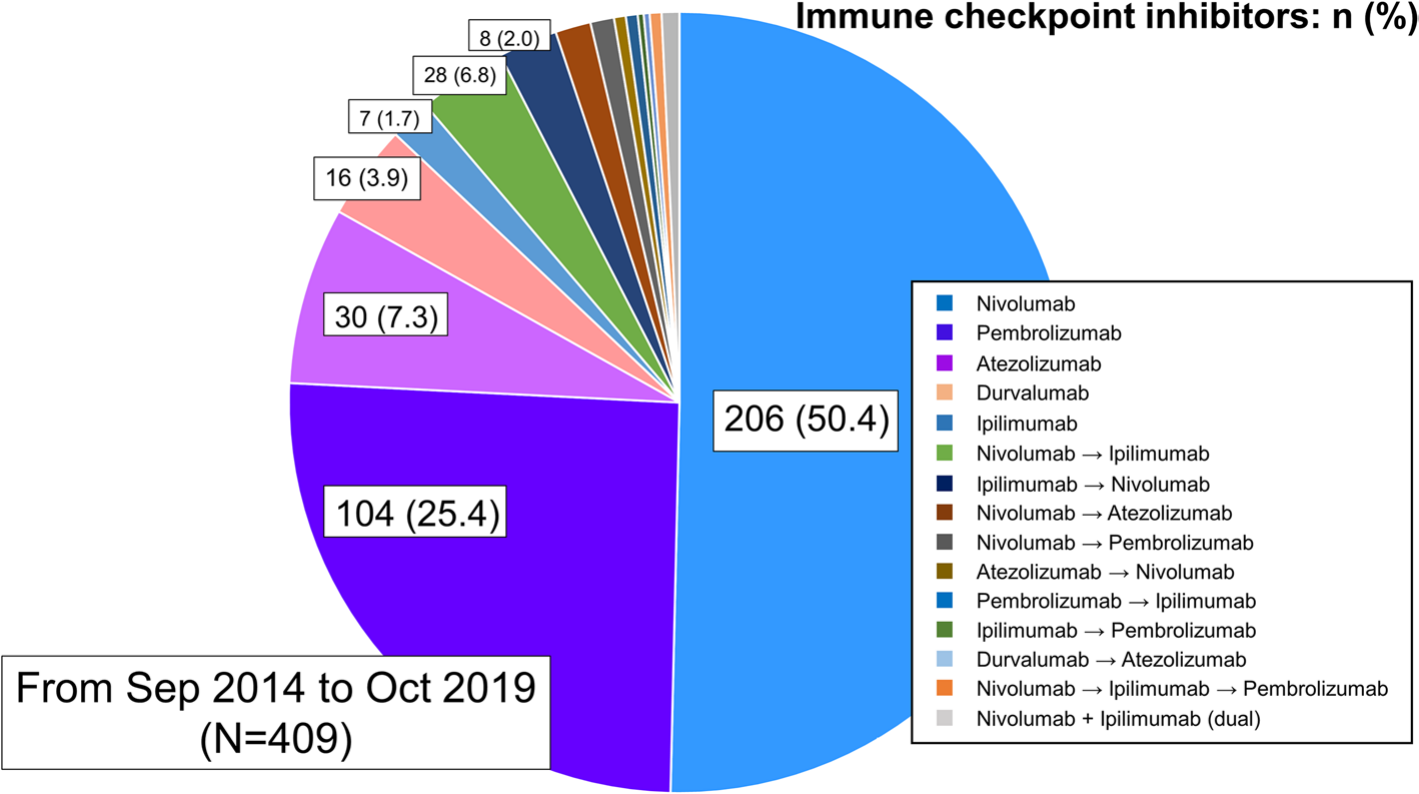
All types and the number of immune checkpoint inhibitors (ICIs). Nivolumab was the most commonly used ICI, followed by pembrolizumab and atezolizumab. Forty-three patients experienced a change in the type of ICI they were administered, and three patients received dual ICI therapy

During the study period, 412 patients with cancer received their first ICI administration. Of these patients, three who lacked adequate data were excluded. Thus, 409 patients were investigated in this study. Figure 1 shows the type of ICI administered. Nivolumab was the most commonly used ICI, followed by pembrolizumab and atezolizumab, and three patients received a dual therapy. The patient characteristics are presented in Table 1. The median age was 69 years, and 121 (29.6%) patients were female. In total, 162 (39.6%), 74 (18.1%), and 72 (17.6%) patients had a medical history of hypertension, diabetes mellitus, and dyslipidemia, respectively, and 69 (16.9%) patients had a history of any cardiovascular disease. Furthermore, 34 (8.3%), 24 (5.9%), 5 (1.2%), 8 (2.0%), and 3 (0.7%) patients had a history of coronary artery disease, arrhythmia, heart failure, venous thromboembolism, and pericardial disease, respectively. Of the 69 patients, 57 (82.6%) patients were followed up by cardiologists or their family physicians, and 48 (69.6%) patients continued to receive medication for cardiovascular comorbidities during ICI therapy. The median time from the onset of cardiovascular disease to the start of ICI therapy was 656 (314–1366) days.

**Table 1:** Patient characteristics at baseline

	All (*n* = 409)	irAEs ( +) (*n* = 138)	irAEs (-) (*n* = 271)	*P* value
Age, years	69 (60–74)	70 (60–74)	69 (60–74)	0.32
Gender (male)	288 (70.4)	94 (68.7)	194 (71.6)	0.49
BMI, kg/m^2^	20.9 (18.1–23.4)	21.7 (18.5–24.0)	20.5 (17.9–23.2)	0.04
Pulse rate, bpm	82 (73–93)	81 (71–91)	84 (74–94)	0.12
Systolic blood pressure, mmHg	120 (107–134)	123 (111–134)	118 (106–133)	0.14
Diastolic blood pressure, mmHg	72 (64–81)	72 (64–81)	72 (64–82)	0.70
**Cancer type**				
Non-small cell lung cancer	170 (41.6)	59 (42.8)	111 (41.0)	0.75
Malignant melanoma	82 (20.0)	42 (30.4)	40 (14.8)	< 0.01
Renal cancer	43 (10.5)	20 (14.5)	23 (8.5)	0.09
Stomach cancer	35 (8.6)	4 (2.9)	31 (11.4)	0.003
Pharyngeal cancer	21 (5.1)	3 (2.2)	18 (6.6)	0.059
Paranasal cancer	7 (1.7)	2 (1.4)	5 (1.8)	1
Tongue cancer	7 (1.7)	1 (0.7)	6 (2.2)	0.43
Bladder cancer	6 (1.5)	1 (0.7)	5 (1.8)	0.67
Mesothelioma	5 (1.2)	0 (0)	5 (1.8)	0.17
Other cancer	33 (8.1)	6 (4.3)	27 (10.0)	0.056
**Comorbidity**				
Hypertension	162 (39.6)	60 (43.5)	102 (37.6)	0.29
Diabetes mellitus	74 (18.1)	27 (19.6)	47 (17.3)	0.59
Dyslipidemia	72 (17.6)	31 (22.5)	41 (15.1)	0.075
**Cardiovascular history**				
Coronary artery disease	34 (8.3)	15 (10.9)	19 (7.0)	0.19
Arrythmia	24 (5.9)	11 (8.0)	13 (4.8)	0.27
Heart failure	5 (1.2)	2 (1.4)	3 (1.1)	1
Venous thrombosis	8 (2.0)	0 (0)	8 (3.0)	0.06
Pericardial disease	3 (0.7)	0 (0)	3 (1.1)	0.55
**Medication**				
ACE-I/ARB	81 (19.8)	35 (25.4)	47 (17.3)	0.067
Beta-blockers	34 (8.3)	15 (10.9)	19 (7.0)	0.26
Ca-channel blockers	118 (28.9)	43 (31.2)	75 (27.7)	0.49
Statins	57 (13.9)	23 (16.7)	34 (12.5)	0.29
Diuretics	28 (6.8)	6 (4.3)	27 (10.0)	0.21
**Laboratory measurements**				
TP, mg/dL	6.8 (6.3–7.1)	6.8 (6.5–7.0)	6.7 (6.3–7.2)	0.37
Alb, mg/dL	3.7 (3.3–4.0)	3.8 (3.4–4.1)	3.7 (3.2–4.0)	0.01
Cre, mg/dL	0.79 (0.65–1.00)	0.78 (0.64–1.00)	0.79 (0.65–1.00)	0.91
CRP, md/dL	0.42 (0.10—1.80)	0.23 (0.07—1.12)	0.56 (0.13 – 2.28)	0.004
WBC, /10^3^	6.0 (4.9—7.7)	5.9 (4.9 – 7.3)	6.0 (4.9—7.8)	0.44
Hb, mg/dL	11.8 (10.4–13.0)	12.0 (10.9–13.2)	11.7 (10.3–12.9)	0.033
**Chest radiography**				
CTR, %	46.5 (42.6–50.1)	46.8 (42.8–50.2)	46.3 (42.5–50.1)	0.56

*irAEs* immune-Related Adverse Events, *BMI* body mass index, *ACE-I* angiotensin-converting-enzyme inhibitor, *ARB* Angiotensin II Receptor Blocker, *TP* total protein, *Alb* albumin, *Cre* creatinine, *CRP* C-reactive protein, *WBC* white blood cell, *Hb* hemoglobin, *CTR* cardiothoracic ratio

When divided into two groups according to the presence or absence of irAEs, no significant intergroup differences were found in terms of age, sex, or comorbidity. The irAE ( +) group had a significantly higher proportion of patients with melanoma and a lower proportion of patients with stomach cancer than the irAE ( −) group. Moreover, body mass index and albumin and hemoglobin levels were significantly higher and c-reactive protein levels were lower in the irAE ( +) group than in the irAE ( −) group.

## Summary of non-cardiovascular and cardiovascular irAEs

**Fig. 2:**
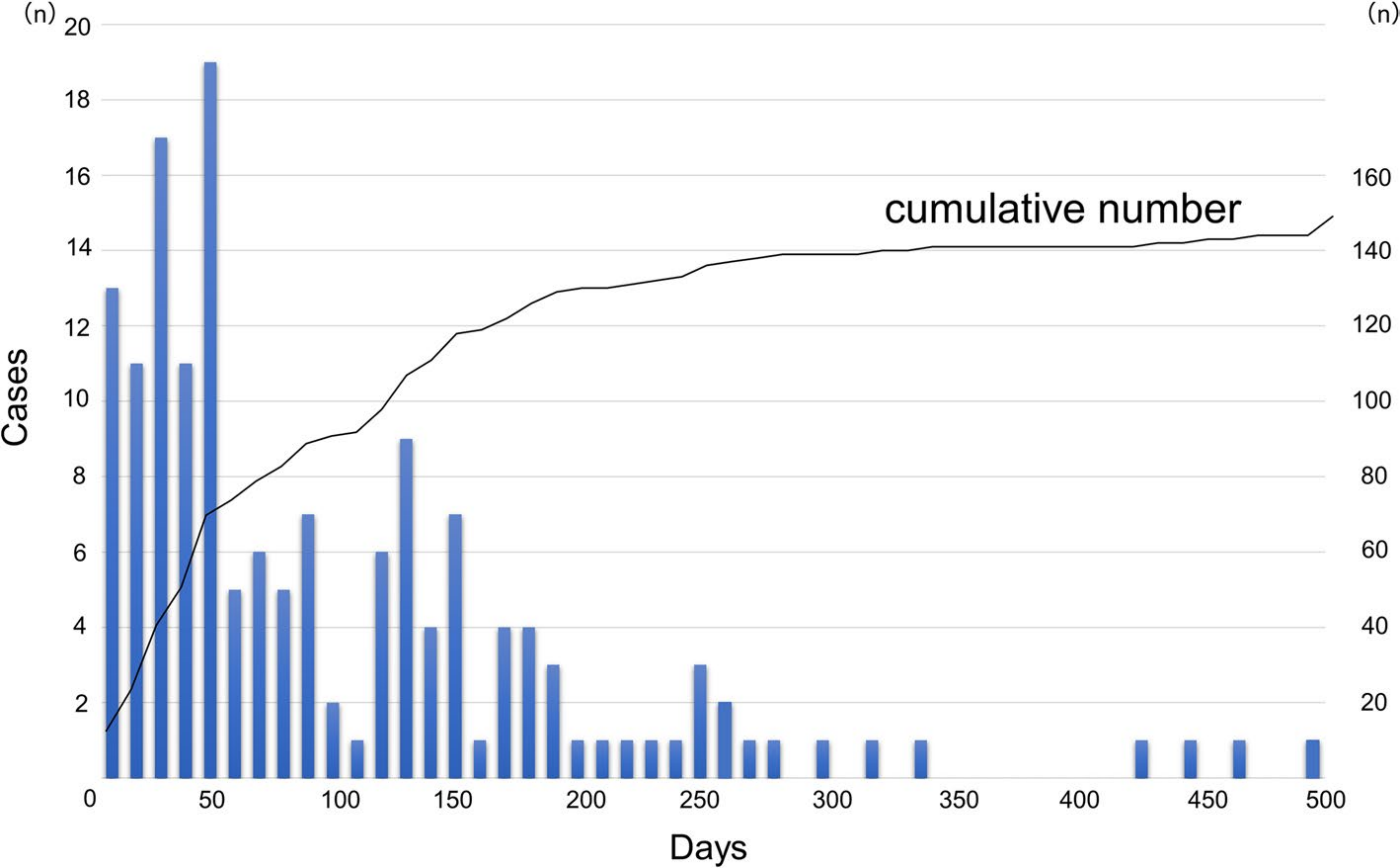
Time to the onset of immune-related adverse events (irAEs) after the first immune checkpoint inhibitor (ICI) administration. The median time from the first ICI administration to the onset of an irAE was 77 (31–168) days

A total of 159 irAEs (grades 2–4) occurred in 138 (33.7%) patients, of whom 20 had multiple irAEs. The details of the irAEs classified according to ASCO guidelines are shown in Table 2. Endocrine toxicities, including destructive thyroiditis and hypophysitis, were the most common irAEs, followed by gastrointestinal toxicities such as colitis and liver dysfunction. Figure 2 shows the time to irAE onset after the initial administration of ICIs. The median length of time from the first ICI administration to the onset of the irAE was 71 (28–161) days. Myocardial vasculitis developed 263 days after the first ICI administration, and fulminant myocarditis developed 495 days after the first ICI administration. Furthermore, 14 (3.4%) patients experienced some cardiovascular irAEs after ICI administration and required consultation with a cardiologist (Table 3). Arrhythmias were the most common of these irAEs, and cardiologist intervention was required by 3 patients with angina pectoris, 2 with pericardial effusion, 1 with pulmonary embolism, and 1 with heart failure. Fulminant myocarditis developed in one patient with malignant melanoma, and myocardial vasculitis developed in one patient with melanoma. Among the 14 patients, 9 (64.3%) with cardiovascular irAEs presented with prior grade 2–4 irAEs at other organs, and the median length of time from non-cardiovascular irAEs to the onset of cardiovascular irAEs was 74 (58–246) days. We compared cardiovascular irAEs in patients with and without prior non-cardiovascular irAE, the incidence rate of cardiovascular irAEs was significantly higher in patients with prior non-cardiovascular irAEs (*P* = 0.017) (Table 4).

**Table 2:** Immune-related adverse events (Grade 2–4)

	All	G2	G3	G4
**1.0 Skin toxicities**				
Rash/Dermatitis	18 (13.0)	17 (12.3)	1 (0.7)	0 (0)
**2.0 Gastrointestinal toxicities**				
Colitis	17 (12.3)	11 (8.0)	6 (4.3)	0 (0)
Hepatitis	17 (12.3)	8 (5.8)	7 (5.1)	2 (1.4)
**3.0 Lung toxicities**				
Pneumonitis	29 (21.0)	21 (15.2)	8 (5.8)	0 (0)
**4.0 Endocrine toxicities**				
Hyperthyroidism	35 (25.4)	33 (23.9)	2 (1.4)	0 (0)
Adrenal insufficiency	3 (2.2)	3 (2.2)	0 (0)	0 (0)
Pituitary hypophystis	16 (11.6)	12 (8.7)	4 (2.9)	0 (0)
Diabetes	2 (1.4)	0 (0)	1 (0.7)	1 (0.7)
**5.0 Musculoskeletal toxicities**				
Arthritis	1 (0.7)	1 (0.7)	0 (0)	0 (0)
Myositis	1 (0.7)	1 (0.7)	0 (0)	0 (0)
**6.0 Renal toxicities**	0 (0)	0 (0)	0 (0)	0 (0)
**7.0 Nervous system toxities**				
Optic neuritis	2 (1.4)	2 (1.4)	0 (0)	0 (0)
**8.0 Hematologic toxicities**				
Autoimmune hemolytic anemia	3 (2.2)	0 (0)	3 (2.2)	0 (0)
Lymphopenia	1 (0.7)	0 (0)	1 (0.7)	0 (0)
**9.0 Cardiovascular toxicities**				
Myocarditis	1 (0.7)	0 (0)	0 (0)	1 (0.7)
Vasculitis	1 (0.7)	0 (0)	1 (0.7)	0 (0)
Pericardial effusion	2 (1.4)	0 (0)	2 (1.4)	0 (0)
Arrhythmia	5 (3.6)	0 (0)	4 (2.9)	1 (0.7)
Angina pectoris	3 (2.2)	0 (0)	2 (1.4)	1 (0.7)
Heart failure	1 (0.7)	0 (0)	0 (0)	1 (0.7)
Pulmonary embolism	1 (0.7)	0 (0)	0 (0)	1 (0.7)
**10.0 Ocular toxicities**	0 (0)	0 (0)	0 (0)	0 (0)

**Table 3:** Summary of cardiovascular irAEs

Case	Cardiovascular complications	Age (years), sex	Malignancy	ICI	Days from first ICI administration to cardiovascular irAEs	Days from non-cardiovascular irAEs to cardiovascular irAEs	Management	Outcome
1	Paf	66, M	Renal cancer	Nivolumab	64	15	Pilsicainide	Survived
2	Paf	71, M	NSCLC	Pembrolizumab	35	25	Anticoagulant therapy	Survived
3	AFL	44, F	NSCLC	Nivolumab	4	(-)	Bisoprolol	Cancer death
4	PSVT	72, F	NSCLC	Nivolumab	89	65	Bisoprolol	Cancer death
5	VT	71, M	Melanoma	Pembrolizumab	6	(-)	Catheter ablation	Survived
6	AP	74, M	NSCLC	Nivolumab	201	(-)	PCI	Survived
7	AP	79, M	NSCLC	Nivolumab	1102	986	Nitrous acid	Survived
8	VSP	58, M	Melanoma	Pembrolizumab	294	204	Nitrous acid	Survived
9	Pericardial effusion	45, M	NSCLC	Pembrolizumab	59	58	Pericardial drainage	Cancer death
10	Pericardial effusion	45, M	NSCLC	Pembrolizumab	253	246	Pericardial drainage	Cancer death
11	PE	77, F	NSCLC	Pembrolizumab	161	(-)	Anticoagulant therapy	Survived
12	HF	71, M	NSCLC	Nivolumab	72	(-)	Diuretics	Cancer death
13	Fulminant myocarditis	59, M	Melanoma	Ipilimumab → Nivolumab	495	418	MCS + PSL pulse	Survived from myocarditis, Cancer death
14	Myocardial vasculitis	79, F	Melanoma	Pembrolizumab	263	74	PSL	Survived

*ICI* immune checkpoint inhibitor, *Paf* paroxysmal atrial fibrillation, *AFL* atrial flutter, *PSVT* paroxysmal supraventricular tachycardia, *VT* ventricular tachycardia, *AP* angina pectoris, *VSP* vasospastic angina, *PE* pulmonary embolism, *HF* heart failure, *NSCLC* non-small cell lung cancer, *PCI* percutaneous coronary intervention, *MCS* mechanical circulatory support, *PSL* prednisolone, Other abbreviations as in Table 1

**Table 4:** Cardiovascular irAEs in patients with and without prior non-cardiovascular irAEs

	All (*n* = 409)	Prior non-cardiovascular irAEs ( +) (*n* = 133)	Prior non-cardiovascular irAEs (-) (*n* = 276)	*P* value
Cardiovascular irAEs	14 (3.4)	9 (6.8)	5 (1.8)	0.017

Abbreviations as in Table 1

### Kaplan–Meier survival curves for all-cause mortality

**Fig. 3:**
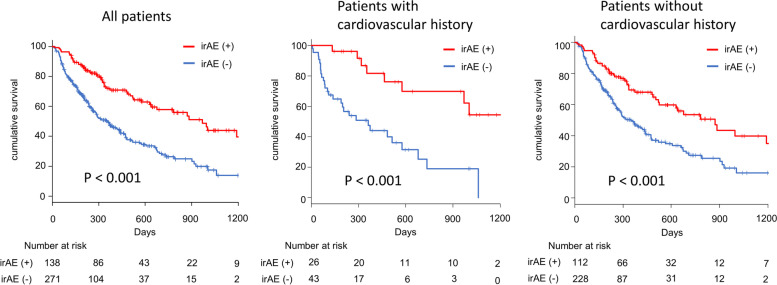
Kaplan–Meier survival analysis for all-cause mortality. The prognosis of patients with immune-related adverse events (irAEs) was significantly better than that of patients without irAEs (*P* < 0.001). This was also detected in patients with a cardiovascular history (*P* < 0.001) and in those without a cardiovascular history (*P* < 0.001)

The median follow-up period was 278 (152–508) days. The comparison of the prognoses between patients who did and did not develop irAEs showed that the prognosis was significantly better for patients with irAEs than for patients without irAEs (*P* < 0.001) (Fig. 3). Furthermore, this tendency was detected in patients with (*P *< 0.001) and without a cardiovascular history (*P* < 0.001). The landmark analysis revealed that even after excluding patients who died within 3 months the prognosis was better for patients with irAEs than for those without irAEs regardless of their cardiovascular history (Supplemental Figure S1).

### Predictors of all-cause mortality

In order to evaluate the prognostic impact of the occurrence of irAEs, we performed cox proportional hazard analysis (Table 5). The occurrence of an irAE and serum albumin level were independent predictors after adjusting for age, sex, body mass index, hemoglobin, C-reactive protein, hypertension, diabetes mellitus, dyslipidemia, and cardiovascular history (occurrence of irAE: HR 0.450, 95% CI 0.322–0.630, *P* < 0.001; serum albumin level: HR 0.438, 95% CI 0.308–0.623, *P* < 0.001) (Table 5). After excluding patients who died within 3 months, the occurrence of an irAE, serum albumin level were also independent predictors of all-cause mortality (Supplemental Table S1).

**Table 5:** Cox proportional hazards regression analysis for all cause mortality, multivariate model

	HR	95% CI	*P* value
Age, per 1 year	0.997	0.983–1.011	0.66
Sex, female	1.240	0.894–1.721	0.20
BMI, per 1 kg/m^2^	0.979	0.942–1.019	0.30
Albumin, per 1 g/dL	0.438	0.308–0.623	< 0.001
Hemoglobin, per 1 g/dL	1.061	0.963–1.169	0.23
CRP, per 1 mg/dL	1.012	0.975–1.050	0.53
Hypertension	0.936	0.678–1.292	0.69
Diabetes mellitus	1.019	0.698–1.487	0.92
Dyslipidemia	1.107	0.733–1.672	0.63
Cardiovascular history	1.150	0.756–1.643	0.58
IrAEs	0.450	0.322–0.630	< 0.001

*HR* hazard ratio, *CI* confidence interval, Other abbreviations as in Table 1

## Discussion

We retrospectively analyzed 409 patients who received ICI therapy, and our results confirmed that patients with irAEs had a better prognosis than those without irAEs regardless of their cardiovascular history. Furthermore, cardiovascular irAEs, which require drug therapy and/or invasive treatment after ICI administration, occurred in 14 (3.4%) patients, and cardiovascular irAEs occurred more frequently in patients with prior non-cardiovascular irAEs. Our results demonstrated that irAEs over grade 2 was a stronger prognostic factor than cardiovascular history.

IrAEs are pathologically characterized by macrophagic and lymphocytic infiltration into the normal tissues and result from the unintended effects of ICI-induced activation of the immune system. Since 2015, various studies have reported that irAEs are associated with a longer prognosis regardless of the treatment regimen [5, 6]. Shimozaki et al. reported that the development of multiple irAEs was associated with longer survivals than a single irAE [19]. Conversely, Naqash et al. reported the negative impact of irAE-related treatment discontinuation on survival [20]. Inhibition of PD-1, PD-L1 and CTLA-4 activates not only tumor-specific T cells but also autoimmunity, and tumor-specific neoplastic antigens and normal tissue antigens may be cross-reactive. These mechanisms may be related to the relation between the occurrence of irAEs and good clinical response, although the precise mechanism has not been fully uncovered [21].

Although several previous reports have demonstrated the relationship between ICI therapy and adverse cardiovascular events [22, 23], no studies have examined the prognostic effects of the coexistence of cardiovascular disease. We speculated the following reasons for the positive impact of irAEs regardless of cardiovascular history: 1) most of cardiovascular comorbidities were stable conditions that more than one year had passed from the onset, and 82.6% of patients with cardiovascular history were followed by cardiologists or family physicians during ICI therapy, 2) fatal cardiovascular irAEs such as myocarditis are infrequent, and in this study, the myocarditis occurred during hospitalization, allowing for prompt therapeutic intervention, and 3) ICIs were used for cancers in which the primary lesion was unresectable and for recurrent or irreversible cancers; therefore, the prognosis of cancer itself was poor.

Similar to Liew et al., who reported that rheumatic irAEs were associated with other non-rheumatic irAEs [24], we found that non-cardiovascular irAEs may increase the incidence of cardiovascular irAEs. In underlying pathophysiology, the main hypothesized mechanism of ICI-induced cardiotoxicity (cardiovascular irAEs) is the extreme reaction of cytotoxic T-cells, which leads to the increased activity of inflammatory and non-inflammatory cytokines. This toxicity may involve any region in the heart, and cytokine storms directly damage the myocardium, pericardium, electrical circuit, endothelial cells, and coagulation function, leading to the destabilization of atherosclerotic lesions. The most representative cardiovascular irAE is myocarditis. Mahmood et al. reported a 1.14% prevalence of ICI-related myocarditis in a multicenter observational study, and fulminant myocarditis was noted in 0.17% of all patients [25]. The median time to onset of myocarditis post ICI administration has been reported as approximately 30 days in most studies [25, 26], although some have indicated that it can occur at any time [27, 28]. In fact, myocardial vasculitis and fulminant myocarditis developed 263 and 495 days after the first administration of ICIs, respectively [8, 29]. Furthermore, in both cases, grade 2 irAEs had been detected in other organs before those conditions occurred (Table 3). At times, it can be difficult to definitively diagnose cardiovascular irAEs, but it should be recognized that ICIs cause cardiovascular complications such as arrhythmia and acute coronary syndrome more frequently than previously reported.

The number of cancer patients who have survived because of rapid advances in treatment drugs has been increasing. In a double-blind, phase three, epoch-making trial of dual ICI therapy, 58% of advanced melanoma patients treated with nivolumab and ipilimumab combination therapy were still surviving at three years [30]. Because the number of patients receiving ICI treatment for long periods has been increasing, it is necessary to pay attention to possible cardiovascular disease. Furthermore, stably controlling cardiovascular comorbidities and preventing fatal cardiovascular events may improve prognoses in patients who have already developed irAEs in other organs.

## Limitations

Our study has some limitations. First, this was a single-center study with a small number of patients, and there was no comparison group such as cancer patients without ICI therapy. Second, we obtained the information retrospectively from medical records, and we did not have a standardized system to perform routine examinations for all patients, such as electrocardiogram, echocardiography, or myocardial biomarkers before and after ICI administration; considering this we may not have investigated some cardiovascular disorders that did not cause major clinical problems. Third, because the follow-up protocol for patients with cardiovascular history was not standardized, we cannot deny the possibility that differences of follow-up affected the results. Fourth, although ICI discontinuation and post ICI therapy would affect prognosis, these factors could not be considered in the statistical analysis. Finally, although it is difficult to exclude the possibility that a longer prognosis increases the rate of irAEs, we statistically showed that irAEs remained an independent predictor.

## Conclusions

Although cardiovascular irAEs may be related to prior non-cardiovascular irAEs under ICI therapy, the occurrence of irAEs had a better prognostic impact and this tendency was not affected by cardiovascular history.

## Supplementary Information

**Additional file 1: Supplemental Figure S1.** Landmark analysis, excluding patients who died within 3 months. Kaplan–Meier survival analysis for all-cause mortality. The prognosis of patients with immune-related adverse events (irAEs) was significantly better than that of patients without irAEs (*P* < 0.001). This was also detected in patients with a cardiovascular history (*P* = 0.014) and in those without a cardiovascular history (*P* = 0.001).

**Additional file 2: Supplemental Table S1.** Cox proportional hazards regression analysis for all cause mortality, excluding patients who died within 3 months, multivariate model

## Data Availability

The datasets used and/or analyzed during the current study are available from the corresponding author on reasonable request.

## Abbreviations

ICI(s): Immune checkpoint inhibitor(s)
irAE(s): Immune-related adverse event(s)
PD-1: Programmed cell death-1
PD-L1: Programmed death-ligand-1
CTLA-4: Cytotoxic T-lymphocyte antigen-4
ASCO: American Society of Clinical Oncology
