# Supplementary figures and images for: Prognostic impact of immune-related adverse events on patients with and without cardiovascular disease: a retrospective review
Source: Cardiooncology. 2021 Jul 6;7:26. doi: 10.1186/s40959-021-00112-z (PMC8259377; doi:10.1186/s40959-021-00112-z)

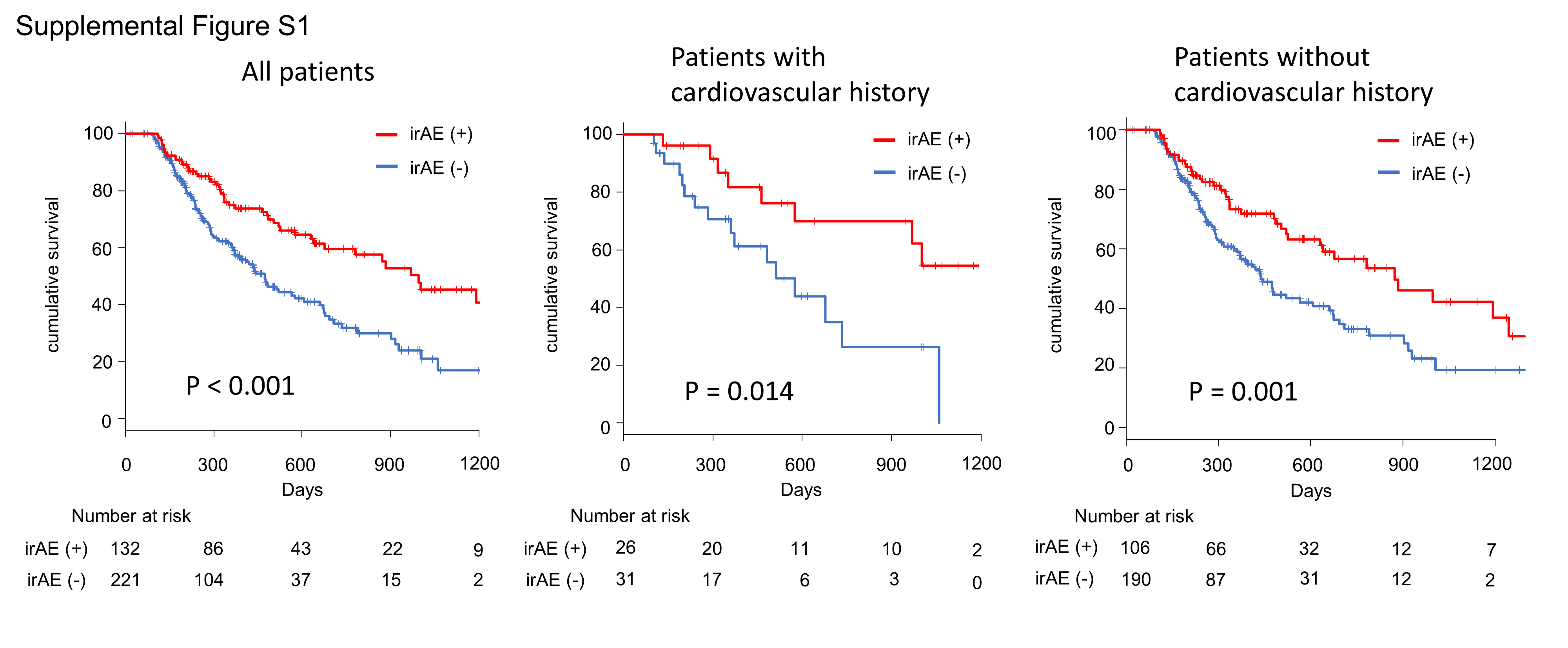

Supplement: Supplementary file 1 — Additional file 1: Supplemental Figure S1. Landmark analysis, excluding patients who died within 3 months. Kaplan–Meier survival analysis for all-cause mortality. The prognosis of patients with immune-related adverse events (irAEs) was significantly better than that of patients without irAEs (P < 0.001). This was also detected in patients with a cardiovascular history (P = 0.014) and in those without a cardiovascular history (P = 0.001). [file 40959_2021_112_MOESM1_ESM.tif]
