# Supplementary material for: Prognostic impact of immune-related adverse events on patients with and without cardiovascular disease: a retrospective review
Source: Cardiooncology. 2021 Jul 6;7:26. doi: 10.1186/s40959-021-00112-z (PMC8259377; doi:10.1186/s40959-021-00112-z)
Supplement: Supplementary file 2 — Additional file 2: Supplemental Table S1. Cox proportional hazards regression analysis for all cause mortality, excluding patients who died within 3 months, multivariate model [file 40959_2021_112_MOESM2_ESM.docx]

| **Supplemental Table S1. Cox proportional hazards regression analysis for all cause mortality, excluding patients who died within 3 months, multivariate model** | | | |
| --- | --- | --- | --- |
|  | HR | 95% CI | P value |
| Age, per 1year | 0.998 | 0.982-1.014 | 0.80 |
| Sex, female | 1.127 | 0.763-1.663 | 0.55 |
| BMI, per 1kg/m^2^ | 0.978 | 0.933-1.025 | 0.35 |
| Albumin, per 1g/dL | 0.591 | 0.378-0.923 | 0.02 |
| Hemoglobin, per 1g/dL | 0.986 | 0.879-1.060 | 0.81 |
| CRP, per 1mg/dL | 0.999 | 0.951-1.050 | 0.98 |
| Hypertension | 0.894 | 0.615-1.301 | 0.56 |
| Diabetes mellitus | 1.135 | 0.741-1.737 | 0.56 |
| Dyslipidemia | 1.151 | 0.724-1.830 | 0.55 |
| Cardiovascular history | 0.869 | 0.546-1.383 | 0.55 |
| IrAEs | 0.519 | 0.357-0.754 | < 0.001 |
| HR = hazard ratio; CI = confidence interval; Other abbreviations as in Table 1. | | | |
